# Reverse Design of High Strength and High Modulus Epoxy Resin Systems Through Computational Modeling with Experimental Validation

**DOI:** 10.3390/polym17091214

**Published:** 2025-04-29

**Authors:** Yilin Tang, Shipeng Zhu, Boya Zhang, Haozhong Lv, Jingshu Wu, Yunhua Yang, Ben Zhang, Jianli Gao

**Affiliations:** Key Laboratory of Advanced Functional Composites, Aerospace Research Institute of Materials & Processing Technology, Beijing 100076, China; tangyl@163.com (Y.T.); boyaz123@163.com (B.Z.); lvhaozhong703@163.com (H.L.); wujingshu12@126.com (J.W.); zhangben9725@163.com (B.Z.); 13683316562@163.com (J.G.)

**Keywords:** epoxy resin, mechanical properties, materials genome approach, formulation design

## Abstract

High-strength and high-modulus epoxy resins are key elements for preparing carbon-fiber-reinforced polymer composites, which play an irreplaceable role in aerospace. In this study, five optimal epoxy systems were developed utilizing the reverse design strategy. The reverse design strategy was based on the ideal resin and curing agent structures offered by the AI polymer platform, and the rules were summarized to create an optimum resin formulation. The formulations used m-phenylenediamine (MPD) as the principal curing agent, which was modified with 10 wt% diethyltetramethylenediamine (DETDA), 10 wt% 4,4′-diaminodiphenylmethane (DDM), or 10 wt% triethylenetetramine (TETA) to establish multiple crosslinking networks. Systematic characterization using differential scanning calorimetry (DSC) and rheological analysis revealed that the optimized activation energy was 55.95–63.42 kJ/mol, and the processing viscosity was ≤500 mPa·s at 80 °C. A stepwise curing protocol (3 h@80 °C, 2 h@120 °C, and 3 h@180 °C) was established to achieve a complete crosslinking network. The results showed that the system with 10% DDM had a tensile strength of 132.6 MPa, a modulus of 5.0 GPa, and a glass transition temperature of 253.1 °C. This work advances the rational design of epoxy resins by bridging molecular architecture with macroscopic performance, offering a paradigm for developing a next-generation matrix tailored to accommodate extreme operational demands in high-end engineering sectors.

## 1. Introduction

Carbon-fiber-reinforced polymer composites (CFRPs) are widely used for manufacturing lightweight structural components in the aerospace industry and are related to their exceptional high specific strength, specific stiffness, and dimensional stability. For instance, resin matrix composites must endure a dynamic impact load of 5 GPa during launch and long-term services at temperatures below −253 °C in rocket liquid hydrogen tanks, necessitating an increased resin modulus to maintain structural integrity. In a similar vein, satellite antenna reflectors must maintain deformation control of less than 0.01 mm/m throughout temperature cycles between −180 °C and +150 °C in order to ensure signal accuracy. This criterion is similarly met by increasing the resin modulus to lessen the distortion caused by heat.

Recently, the development of next-generation carbon fiber (CF) provides a solid foundation for preparing CFRPs with exceptional mechanical properties under extreme service conditions. For example, the tensile strength and modulus of T1100 CF reach as high as 7 GPa and 324 GPa, respectively [1,2]. However, commercially available epoxy resin generally exhibits a tensile strength of about 80 MPa and a modulus ranging from 3 to 3.5 GPa, which are significantly inferior to those of advanced CFs. As a result, the overall performance, especially the compressive strength of CFRPs, is constrained by the properties of the resin matrix, necessitating the development of a high strength and high modulus epoxy resin matrix with the aim of fully unleashing the performance of composites with advanced CFs.

Tensile modulus and tensile strength are two key metric parameters that distinguish advanced epoxy resins from their conventional counterparts. The tensile modulus of the matrix directly affects the transverse support to CFs, enabling them to withstand lateral stress. Additionally, the tensile strength of epoxy resin is critical in complex stress fields, which determines the ultimate load-bearing capacity of the composite [3,4]. These properties of the resin matrix are essential for optimizing the performance of CFRPs. Conventional methods for modifying epoxy resins typically focus on single-component modifications or the optimization of stoichiometric ratios to improve their performance. However, a trade-off remains between the tensile strength and tensile modulus. For example, although adding flexible chain segments improves the toughness of the matrix, the tensile modulus is negatively affected; while incorporating rigid groups increases the tensile modulus, the matrix becomes brittle, illustrating a clear “seesaw” effect between these properties [5,6,7,8,9,10].

Multi-component synergistic enhancement strategies have shown promise in designing high-performance CFRPs. Yet, the optimization processes largely depend on empirical trial-and-error or orthogonal experiments, which are time-consuming, costly, and limited in scope [11]. The advent of the materials genome approach (MGA) offers a new framework for the precise design of epoxy resins. Combining this with the machine learning (ML) technique, MGA provides deeper insights into the structure–performance relationship and enables the efficient design of new materials [12,13,14,15]. Building on this foundation, the AI polymer platform, developed by the East China University of Science and Technology, integrates the MGA with ML to establish a comprehensive multi-scale modeling framework. The framework is able to provide molecular genome analysis, quantitative structure–property prediction, virtual design, and high-throughput screening, thereby enabling the efficient and inverse design of epoxy resins [16]. The accuracy of using this platform to predict the viscosity, glass transition temperature (T_g_), and modulus has been proven in previous studies. Notably, the PSNP-MV resin (with a T_g_ of 608 °C) and 20M28 epoxy resin (achieving a moisture absorption error of only 0.02%) showed consistency between the predicted and experimental results [17,18].

In this work, An AI-assisted reverse design workflow was employed, where the AI polymer platform pre-screened candidate formulations based on target mechanical properties, followed by experimental validation. The influence of the molecular structure and compositions of curing agents on the viscosity, curing kinetics, thermal properties, and mechanical performance of epoxy resin was systematically investigated. This work provides a facile approach to the design of high-performance epoxy resin, which meets stringent demands in the aerospace, aeronautics, automotive, and construction sectors, among others.

## 2. Materials and Methods

### 2.1. Materials

**Figure 1:**
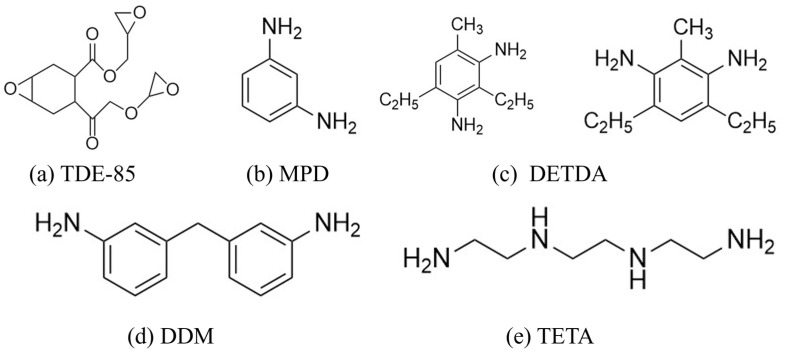
The chemical structure of the epoxy resin and curing agents.

The 4,5-Epoxyhexane-1,2-dicarboxylic acid diglycidyl ester (TDE-85) with an epoxy value of 0.85 eq/100 g was supplied by Tianjin Jingdong Chemical Composites Co., Ltd., (Tianjin, China). m-Phenylenediamine (MPD) was purchased from Shanghai Aladdin Biochemical Technology Co., Ltd., (Shanghai, China). 3,3′-aiaminodiphenylmethane (DDM) was obtained from Sinopharm Group Chemical Reagent Co., Ltd., (Shanghai, China); diethyltetramethylenediamine (DETDA) was supplied by Henan Alpha Chemical Corporation (Zhengzhou, China); and triethylenetetramine (TETA) was provided by Tianjin Yanhai Chemical Corporation (Tianjin, China). The molecular structures of the TDE-85 and curing agents (MPD, DDM, DETDA, and TETA) are shown in Figure 1.

### 2.2. Formulation Design

In order to effectively support the CFs and obtain a composite with excellent compressive strength, we used the AI polymer platform to reserve the design for the resin system, the specific operation for which is given as follows:

(1) Formulation Prediction: The database of the AI polymer platform covers a large amount of structural property data of polymer materials and uses machine learning to construct a structure–performance quantitative prediction model, which realizes the simultaneous prediction of tensile strength and elastic modulus by capturing the constitutive relationship between the topological features of the functional groups and mechanical response. When the target strength (100 MPa) and target modulus (4 GPa) were entered, the platform reverse engineers the resin system to meet the target performance using a virtual design-high-throughput-screening method. The resulting resin formulation is shown in Table 1.
(2) Regularity Analysis: According to Table 1, the essential design principles for the epoxy resin and curing agents are described as follows:
  (a) Multifunctional epoxy groups: Incorporating epoxy resins with multiple functional groups enhances the reactivity, facilitating the formation of a dense crosslinked network that simultaneously improves the tensile strength and tensile modulus.
  (b) Rigid organic structural motifs: Integrating rigid structural elements, such as aromatic rings and biphenyl frameworks, into the molecular structure of resins and curing agents increases their stiffness and resistance to deformation, thereby boosting both the tensile strength and tensile modulus.
  (c) Strong polar interactions: The inclusion of polar functional groups (e.g., hydroxyl and amino) enhances intermolecular hydrogen bonding and dipole–dipole interactions, thereby strengthening interfacial adhesion and enhancing modulus.
(3) Preliminary Formulation: Based on the above screening criteria and principles, TDE-85 was selected as the epoxy matrix and MPD was used as the primary curing agent. TDE-85 was chosen for its trifunctional glycidyl ether structure, which enhances crosslinking density and chain rigidity, leading to improved mechanical properties. In addition, low viscosity favors effective impregnation during the processing of carbon-fiber-reinforced composites. MPD contains a rigid aromatic amine structure, which helps maintain high modulus while controlling curing reaction rates to prevent the formation of defects.
(4) Formulation Optimization: However, a two-component resin system consisting of TDE-85 and MPD may face the following issues:
  (a) Uncontrolled exothermic reactions: The curing profile of a two-component system faces rapid curing and the concentration of internal stress, which likely leads to the formation of defects and the deterioration of mechanical properties.
  (b) High brittleness: The rigid structure of both TDE-85 and MPD leads to a brittle feature of the cured system with limited toughness, which is undesirable for applications in industrial sectors that require both enhanced durability and resistance to damage.
  (c) Poor processability: The viscosity of TDE-85 is suitable for the impregnation of prepregs, but it is still challenging due to the rigidity and high reactivity of the reaction system.

To address the above issues and optimize the curing system, DDM, DETDA, and TETA, which were selected from Table 1 are introduced. In this scenario, DDM has a higher melting point (92~95 °C) when compared with MPD. Thus, the reactivity of DDM is lower than MPD, which enables better control of the curing reaction and facilitates the formation of a dense crosslinking network. DETDA is a liquid curing agent featuring flexible aliphatic chains, and the introduction of DETDA mitigates the brittleness of the cured system by improving the flexibility of chain segments. TETA is also a liquid curing agent with a polyamine moiety. The addition of TETA enables the fine tuning of the crosslinking density through its gradient reactivity which endows the cured system with balanced strength and processability. As a result, the introduction of DDM, DETDA, and TETA helps strike a balance between mechanical properties and processability, addressing the limitations of a two-component cured system and optimizing the overall performance.

### 2.3. Preparation of Epoxy Resin

Firstly, the TDE-85 epoxy resin was heated to 80 °C, and a specified amount of MPD was added in equal proportions to TDE-85. Then, the mixture was stirred for 30 min until the MPD was fully dissolved. Afterward, DDM, DETDA, and TETA were separately added into the TDE-85/MPD system, followed by thorough blending. Then, the above system was subject to vacuum degassing to completely eliminate air bubbles. The resin mixture was poured into a preheated mold at 80 °C and the curing was carried out as per the following protocol: 3 h@80 °C, 2 h@120 °C, and 3 h@180 °C. Compared to the high brittleness of DDM and low heat resistance of TETA, DETDA was chosen for compounding in subsequent experiments. The formulation for the prepared samples is provided in Table 2. The samples were labeled TTM-1, TTM-3 to TTM-5, where the numeric suffixes indicated the change in the DETDA content (TTM-1: 10%, TTM-3: 30%, TTM-5: 50%), while TDM indicated the DDM composite system, and TEA indicated the TETA composite epoxy system.

### 2.4. Characterizations

The viscosity–temperature profile of the resin systems was obtained using an Mcr 102E rheometer (Anton Paar Co., Ltd., Graz, Austria). Fourier transform infrared (FT-IR) spectra were recorded in the range of 400–4000 cm^−1^ using a NICOLET 6700 spectrometer (Thermo Fisher Scientific, Waltham, MA, USA) with a resolution of 4 cm^−1^. Differential scanning calorimetry (DSC) was performed using a Setaram DSC 141 differential scanning calorimeter (DSC, Setaram, Caluire-et-Cuire, France). Samples were heated from 50 to 250 °C at 5, 10, 15, and 20 °C/min, respectively. Dynamic mechanical analysis (DMA) was carried out on rectangular samples with dimensions of 40 × 6 × 2 mm^3^ using a 202-E dynamic mechanical thermal analyzer (Netzsch Instruments, Selb, Germany). The samples were tested in the 3-point-bending loading mode by heating from 50 to 300 °C at 5 °C/min. The tensile properties of the cured samples were tested using an MTS Model E45.105E universal material testing machine (MTS Systems Corporation, Eden Prairie, MN, USA) according to ASTM D638. The tests were conducted at a crosshead speed of 2 mm/min.

## 3. Results

### 3.1. Curing Behavior of Resin Systems

**Figure 2:**
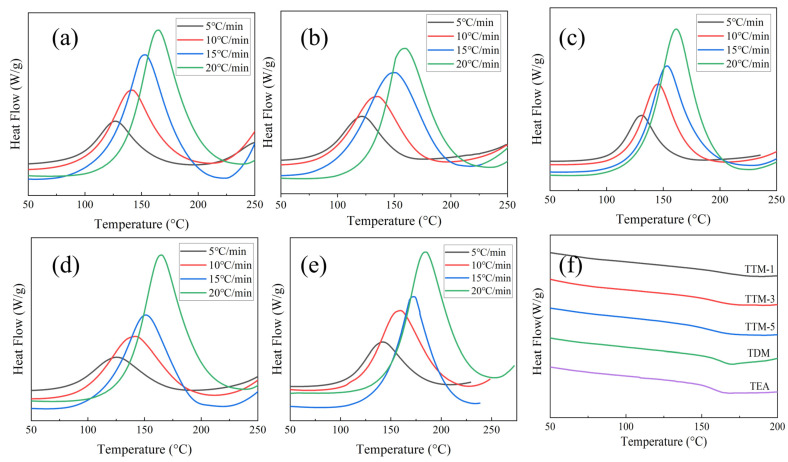
DSC heating curves of various blended resin systems: (**a**) TTM-1; (**b**) TTM-3; (**c**) TTM-5; (**d**) TDM; (**e**) TEA; and (**f**) DSC heating curves of cured resin systems.

To elucidate the curing behavior of the resin systems, DSC measurements were conducted to investigate the heat absorption and exothermic characteristics of various resin formulations. The DSC curves of the blended systems prior to curing are shown in Figure 2. The results indicate that all samples exhibited a single exothermic peak during the curing process, and the peak value shifted to higher temperatures with increasing heating rates. The shift in the exothermic peak towards higher temperatures at higher heating rates is related to the enhanced thermal effect per unit of time. Additionally, both the peak temperature and peak area of exothermic response increased with an increase in the heating rate, which suggested an accelerated reaction rate. As a result, high heating rates resulted in a reduction in the curing time and a high heat release associated with the curing process [19]. The DSC curves of cured samples are presented in Figure 2f. The results show that all samples exhibited a flattened pattern without the presence of exothermic peaks, indicating a complete reaction between the epoxy resin and curing agent.

The apparent activation energy (*E*_a_) that quantifies the energy barrier for the curing reaction was calculated to further understand the curing behavior of blended epoxy resin systems. By employing the Kissinger [20], Ozawa [21], and Crane [22] equations, the essential parameters such as reaction *E*_a_, the number of reaction stages (n) and the front factor can be derived, which is crucial for constructing accurate models to describe the curing process. The equations for the above-mentioned methods are given below:

Kissinger method:

(1) $$\ln\left(\beta /{T_{p}}^{2}\right)=\ln\left(AR/E_{a}\right)-E_{a}/RT_{p}$$

Ozawa method:

(2) $$ln\beta =-1.052E_{a}/RT_{p}$$

Crane method:

(3) $$d(ln\beta )/d\left(1/T_{p}\right)=-(E_{a}/nR+2T_{p})$$

When EanR≫2Tp, then

(4) $$d(ln\beta )/d\left(1/T_{p}\right)=-E_{a}/nR$$

where *β* is the heating rate (°C/min), *T*_p_ is the peak temperature (°C), *R* is the ideal gas constant (8.314 J/(mol·K)), and *E*_a_ is the apparent activation energy (J/mol).

The values of *E*_a_ and n for different blended epoxy resin systems are tabulated in Table 3. The *E*_a_ was calculated by utilizing the heating rate and peak temperature (Table 4). The n was calculated from the Crane equation. The results show that the values of *E*_a_ ranged from 46.39 (TEA) to 62.21 kJ/mol (TTM-5) using the Kissinger method. They reveal that the curing system containing TEA had the smallest *E*_a_ while TTM-5 had the highest *E*_a_. Moreover, the *E*_a_ of the TTM system increased from 51.48 (TTM-1) to 62.21 kJ/mol (TTM-5) with an increase in the DETDA content.

The above results indicate that the TEA-cured system exhibited the lowest average *E*_a_ (46.39 kJ/mol) among the studied systems, indicating its high reactivity and efficient curing performance. This is attributed to its unique molecular structure as an aliphatic amine, as characterized by the three amino groups linked by methylene. This typical structure provides high flexibility and lacks site-blocking effects, enhancing its ability to react with epoxy groups at relatively lower temperatures. In comparison with the other amine curing agents, the reactivity follows the order of aliphatic amine > alicyclic amine > aromatic amine [23], supporting the TEA’s position as the most reactive curing agent among the studied systems.

The TDM system has a greater *E*_a_ (57.71 kJ/mol) than TTM-1 (51.48 kJ/mol), indicating an enhanced energy barrier for the curing reaction. DDM’s stiff bi-phenyl structure (compared to TTM-1’s single benzene ring) encourages stronger intermolecular π-π interactions, resulting in a densely packed pre-cured network that requires more energy to break it. Furthermore, extended conjugation between the amino group and dual benzene rings reduces electron density, lowering the nucleophilicity of amine groups. This electron drawdown effect reduces the reactivity of DDM’s -NH groups, raising the energy barrier for epoxy ring opening.

As the content of DETDA increased, the apparent *E*_a_ of the TTM systems gradually increased. This was attributed to the steric hindrance effect of the methyl and ethyl groups in DETDA, which limited the reactivity of the amino group on the benzene ring and the system required more energy to overcome the potential barrier of the reaction, thus increasing the *E*_a_ of the system.

In summary, compounding systems containing aliphatic amines exhibit the lowest *E*_a_, making them suitable for low-temperature curing processes; on the other hand, the epoxy systems containing aromatic amines needed to overcome higher *E*_a_, making them suitable for medium- to high-temperature curing processes.

### 3.2. FTIR Spectra

**Figure 3:**
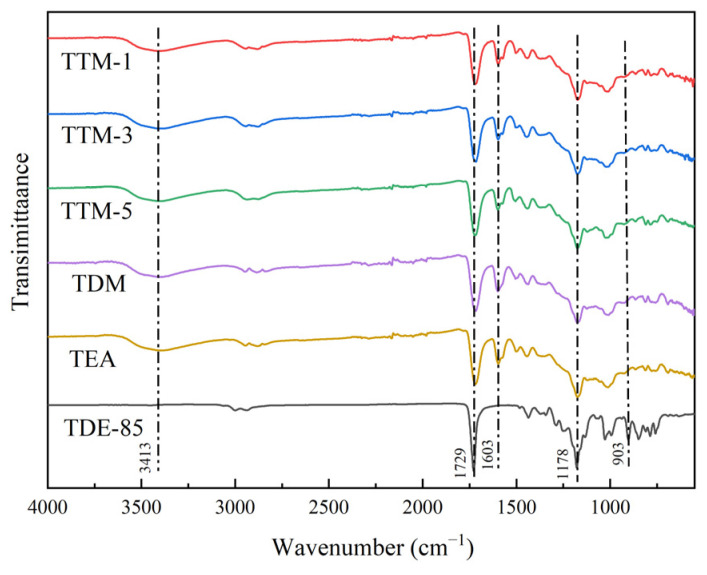
FTIR spectra of TDE-85 epoxy resin with different curing agents.

The FTIR spectra of TDE-85 before and after the curing reaction are presented in Figure 3. Combining this with the DSC results, a comprehensive understanding of the curing process was elucidated. As for the cured systems, a prominent absorption peak at 1603 cm^−1^ was observed, which was attributed to the C=C skeletal stretching vibration of the benzene ring in aromatic curing agents such as DDM and DETDA. Although TETA is an aliphatic amine and does not contain a benzene ring, TEA showed the presence of the absorption peak at 1603 cm^−1^, which was likely related to residual aromatic components from the primary curing agent, MPD.

The complete disappearance of the epoxy group stretching vibration peak at 903 cm^−1^ post-curing confirmed the successful occurrence of the ring-opening reaction between epoxy groups and amino groups, as supported by the DSC data showing the progression of the curing reaction. The reaction also led to a slight decrease in the area of the ether bond (C-O-C) stretching vibration at 1178 cm^−1^ when compared to uncured pure TDE-85. This reduction in the infrared absorption intensity of ether bonds suggested an increase in the crosslinking density, which restricted the mobility of molecular chain segments. The above observation was aligned with the DSC results because a higher *E_a_* was needed for the cured systems to achieve higher crosslinking densities, such as those involving aromatic amines.

The peak at 3413 cm^−1^, which corresponded to the stretching vibration of the hydroxyl group formed through the ring-opening reaction between the epoxy group and the amino group [24], further supported the completion reaction of the cured epoxy systems, as characterized by DSC. Overall, combining the FTIR spectra and DSC results, the progression of the curing reaction, the formation of new functional groups, and the impact of crosslinking density on the system’s energetics were elucidated, which provided valuable insights into the curing process and structural changes during the reaction.

### 3.3. Viscosity Analysis

**Figure 4:**
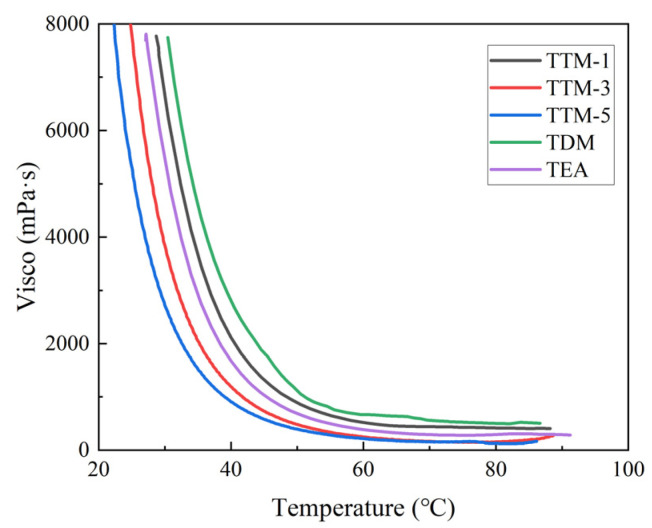
The viscosity of blended epoxy resin systems as a function of temperature.

Viscosity is a critical processing parameter for epoxy resin compounds, as shown in Figure 4. The viscosity of the resin system decreased with the increasing temperature, which was related to the enhanced mobility of molecular chains. At a constant temperature, the TDM system exhibited the highest viscosity, which was attributed to its rigid benzene ring structure and strong intermolecular interactions. In contrast, DETDA and TETA, as well-flowing liquids at room temperature, significantly reduced the viscosity of the resin system. For the TTM system, lower DETDA concentrations initially promoted a rapid reaction between the amino and epoxy groups, forming a relatively homogeneous crosslinking network structure at the early curing stage. However, increasing the DETDA concentration introduced steric hindrance from the diethyl groups, which weakened the intermolecular interactions and reduced free volume, leading to a gradual decrease in viscosity. The above highlights the interplay between the molecular structure, reaction kinetics, and processing properties of epoxy resin systems with different combinations of curing agents.

### 3.4. Thermal Properties

The glass transition temperature (T_g_) is a crucial parameter that characterizes the transition of polymeric materials from a glassy state to a viscoelastic rubbery state, reflecting the mobility threshold of molecular chain segments. DMA was adopted to investigate the T_g_ of different compounding systems, with T_g_ determined by the peak of the loss tangent (tan δ) curve.

**Figure 5:**
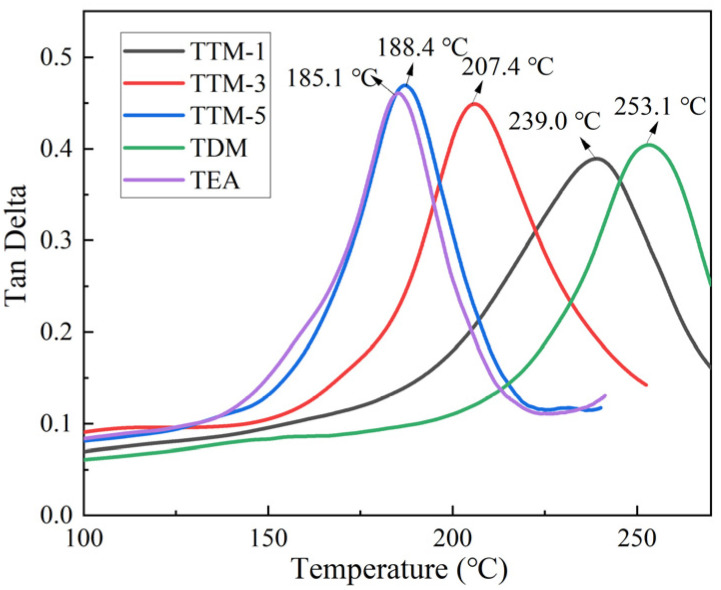
Dynamic mechanical analysis of different compounding systems.

The tan δ curves of different blended systems as a function of temperature are displayed in Figure 5. The results reveal that the TDM system demonstrated the highest T_g_ among the studied systems which was related to its intrinsic rigid benzene ring structure and strong intermolecular π-π interactions, creating a highly crosslinked network that restricted the movement of molecular chains [25].

A higher crosslinking density aligns with the higher *E*_a_s observed in DSC, indicating a greater energy barrier for the curing reaction. In contrast, the TEA system, with a T_g_ of 185.1 °C, was consistent with the lowest *E*_a_ due to the flexible aliphatic chains of TETA. The flexible aliphatic chains exhibited a high degree of chain mobility which reduced the energy required for the curing process.

The TTM systems showed a progressive decrease in T_g_ from 239.0 °C (TTM-1) to 188.4 °C (TTM-5) with the increasing DETDA content, reflecting the influence of flexible side chains and low steric hindrance in DETDA. However, the reduction in the values of T_g_ was inconsistent with the observed increase in *E*_a_. The flexible methyl and ethyl side chains in DETDA increased the free volume within the cured system, allowing for greater mobility of molecular chain segments, even at lower temperatures, exhibiting lower T_g_ values. However, the flexible side chains likely created a steric hindrance, which increased the complexity of the reaction, thereby requiring more energy to overcome the reaction barrier.

The values of volume density for different blended epoxy resin systems were measured by the drainage method, and the results are tabulated in Table 5. The results show that the density of the cured systems ranged from 1.28 to 1.32 g/cm^3^. In general, an increase in the rigidity of the cured systems led to an increase in the crosslinking density, which correlated with a higher T_g_s. The analysis of T_g_ of the blended systems revealed a clear correlation between the molecular structure and crosslinking density, which was aligned with the DSC analysis to gain a comprehensive understanding of curing behavior.

### 3.5. Mechanical Properties

**Figure 6:**
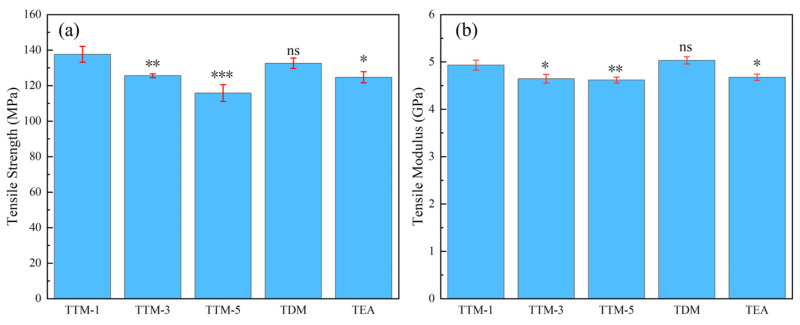
Tensile properties of different blended epoxy resin systems: (**a**) Tensile strength; (**b**) Tensile modulus. (* Statistical significance between groups was determined using Student’s two-tailed *t*-test (α = 0.05), with TTM-1 as the baseline control. Significance levels were denoted as follows: * *p* < 0.05, ** *p* < 0.01, *** *p* < 0.001”).

The mechanical properties including tensile strength and tensile modulus of different blended systems are shown in Figure 6. and Table 5. The results show that the TTM-1 system exhibited a tensile strength as high as 137.7 MPa and a tensile modulus of 4.96 GPa. The TTM-1 system displayed a significantly higher tensile strength when compared to the optimized systems (vs. TTM-3: ** *p* = 0.01; vs. TTM-5: * *p* = 0.004; vs. TEA: * *p* = 0.015). The results showed that the tensile strength of the TTM-1 system was as high as 137.7 MPa and the tensile modulus was 4.96 GPa. This is in agreement with the above DMA results, showing that TTM-1 has a dense crosslinked network. The equilibrium structure of the rigid benzene ring and flexible alkyl chain increased the load-bearing capacity and stress dissipation ability. However, increasing the DETDA content in TTM systems led to a reduction in the crosslinking density, as indicated by a decreasing bulk density from 1.30 (TTM-1) to 1.28 g/cm^3^ (TTM-5) as per the Fox–Flory free volume theory [26]. In conjunction with the viscosity test, a higher content of DETDA reduced the zero-shear viscosity of the resin system, accelerated the early flow, and reduced the final crosslink density. As a result, the tensile strength and tensile modulus of TTM systems experienced a gradual reduction. Specifically, TTM-3 and TTM-5 showed progressively lower tensile strengths of 125.6 and 115.8 MPa, respectively, along with a slightly reduced tensile modulus of 4.67 and 4.61 GPa.

In contrast, the TDM system, with its double benzene ring structure, formed a high-density rigid crosslinking network (1.32 g/cm^3^), resulting in the highest tensile modulus (5.06 GPa) and T_g_ (253.1 °C) among the studied systems. Despite its structural rigidity, TDM’s tensile strength (134.2 MPa) showed no significant difference from TTM-1 (*p* = 0.176), likely due to the stress concentration caused by limited chain mobility under high viscosity. The TEA system, with a tensile strength of 124.7 MPa and a tensile modulus of 4.71 GPa, exhibited relatively lower mechanical properties when compared to TDM and TTM-1, and this was attributed to the flexible aliphatic chains of TETA that reduced the overall rigidity and strength of the crosslinked network. These results establish a mechanistic link between the curing agent structure, thermal property, viscosity, and mechanical performance. Adjusting the balance between the rigid modulus region and flexible energy dissipation region is a key design parameter for customizing high-performance epoxy composites.

In addition to the above observations, Table 5 compares the mechanical and thermal properties of the cured resins with data from the recent literature. A comparative analysis of the data demonstrates the value of using the reverse engineering design method based on the AI polymer platform to develop resins with high modulus and high strength as well as excellent thermal properties.

## 4. Conclusions

In this work, a hybrid epoxy resin system with superior mechanical properties and processability was developed with the aid of the AI polymer platform, as confirmed by the experimental validations. The tensile strength and tensile modulus of the TDE-85 epoxy resin systems with different types of curing agents exceeded 100 MPa and 4.0 GPa, respectively, surpassing the requirements for preparing advanced composites. In addition, the TDM system exhibited a remarkable tensile strength of 132.6 MPa and a tensile modulus of 5.0 GPa along with a T_g_ of 253.1 °C, which is suitable for high-temperature applications. The TEA system achieved a tensile strength of 124.7 MPa and a tensile modulus of 4.7 GPa with a T_g_ of 185.1 °C. This work provides a facile approach to developing high strength and high modulus epoxy resin systems using the reverse design of molecular structure based on material genome and machine learning approach. The “structure-process-performance” framework which was constructed via the AI polymer platform provided a solid foundation for the rational design of a resin system, paving the way for future advancements that require robust performance. Based on the above findings, the preparation of high-performance carbon-fiber-reinforced epoxy composites is underway and will be reported in future work.

## Figures and Tables

**Table 1:** The predicted resin systems with targeted performance by the AI polymer platform.

No.	Epoxy Resin	Curing Agent	Tensile Strength (MPa)	Tensile Modulus (GPa)
1	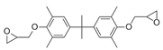	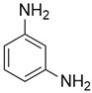	118.2 ± 40.7	4.3 ± 1.5
2	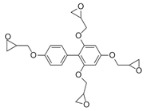	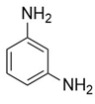	101.5 ± 44.8	4.3 ± 1.6
3	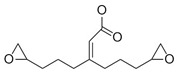	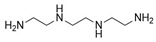	105.3 ± 42.3	4.0 ± 2.1
4	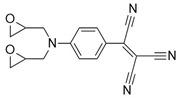	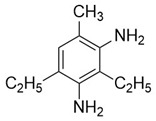	100.5 ± 37.8	4.1 ± 1.4
5	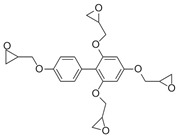	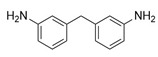	120.2 ± 41.4	4.4 ± 1.5

**Table 2:** The formulation of epoxy resin systems with different curing components.

Sample ID	TDE-85 (g)	MPD (g)	The Secondary Curing Agent			Content of the Secondary Curing Agent (mol%)
			DETDA/g	DDM/g	TETA/g	
TTM-1	100	19.41	4.6			10
TTM-3	100	13.5	9.5			30
TTM-5	100	8.7	14.3			50
TDM	100	19.1		3.9		10
TEA	100	20.0			3.0	10

**Table 3:** Activation energies and reaction stages of various compounding systems.

Method	TTM-1	TTM-3	TTM-5	TDM	TEA
Kissinger *E*_a_ (kJ/mol)	49.33	68.97	60.40	55.47	43.96
Ozawa *E*_a_ (kJ/mol)	53.63	49.35	64.02	59.94	48.81
Average *E*_a_ (kJ/mol)	51.48	59.16	62.21	57.71	46.39
n	0.912	1.140	0.924	0.921	0.903

**Table 4:** Kinetic parameters of various compounding systems.

System	β (°C·min^−1^)	*T*_p_ (K)	ln(β/*T*_p_^2^)	lnβ	10^3^/*T*_p_/K^−1^
TTM-1	5	399.00	−10.36	1.60	2.50
	10	415.87	−9.76	2.30	2.40
	15	428.29	−9.41	2.70	2.33
	20	435.09	−9.16	3.00	2.30
TTM-3	5	394.74	−10.34	1.60	2.53
	10	410.33	−9.73	2.30	2.43
	15	420.15	−9.37	2.70	2.38
	20	432.63	−9.14	3.00	2.31
TTM-5	5	403.69	−10.39	1.60	2.48
	10	417.92	−9.76	2.30	2.39
	15	426.07	−9.40	2.71	2.34
	20	434.06	−9.15	3.00	2.30
TDM	5	404.84	−10.40	1.61	2.47
	10	421.51	−9.79	2.30	2.37
	15	428.18	−9.41	2.71	2.34
	20	438.40	−9.17	3.00	2.28
TEA	5	414.95	−10.45	1.61	2.41
	10	431.02	−9.83	2.30	2.32
	15	445.29	−9.49	2.71	2.25
	20	457.62	−9.26	3.00	2.19

**Table 5:** The mechanical properties, T_g_, and volume density of blended epoxy resin systems.

Blended System/Reference	Tensile Strength (MPa)	Tensile Modulus (GPa)	T_g_ (°C)	Volume Density (g/cm^3^)
TTM-1	137.7 ± 4.5	4.96 ± 0.06	239.0	1.30
TTM-3	125.6 ± 1.1	4.67 ± 0.05	207.4	1.29
TTM-5	115.8 ± 4.7	4.61 ± 0.06	188.4	1.28
TDM	132.6 ± 3.2	5.06 ± 0.07	253.1	1.32
TEA	124.7 ± 3.1	4.71 ± 0.08	185.1	1.28
[8]	134.72	5.04	155.5	-
[15]	86.4	6.46	218.0	-
[18]	132.79	4.49	-	-

## Data Availability

The original contributions presented in this study are included in the article. Further inquiries can be directed to the corresponding authors.
